# Automated wide-line nuclear quadrupole resonance of mixed-cation lead-halide perovskites

**DOI:** 10.5194/mr-6-143-2025

**Published:** 2025-07-16

**Authors:** Jop W. Wolffs, Jennifer S. Gómez, Gerrit E. Janssen, Gilles A. de Wijs, Arno P. M. Kentgens

**Affiliations:** 1 Institute for Molecules and Materials, Radboud Universiteit, Heyendaalseweg 135, 6525 AJ Nijmegen, the Netherlands

## Abstract

Nuclear quadrupole resonance (NQR), a technique related to nuclear magnetic resonance, is extremely sensitive to local crystal composition and structure. Unfortunately, in disordered materials, this sensitivity also leads to very large linewidths, presenting a technical challenge and requiring a serious time investment to get a full spectrum. Here, we describe our newly developed, automated NQR set-up to acquire high-quality wide-line spectra. Using this set-up, we carried out ^127^I NQR on three mixed-cation lead-halide perovskites (LHPs) of the form MA_*x*_FA_1−*x*_PbI_3_ (where MA denotes methylammonium; FA denotes formamidinium; and x= 0.25, 0.50 and 0.75) at various temperatures. We achieve a signal-to-noise ratio of up to ∼400 for lineshapes with a full width at half maximum of ∼2.5MHz acquired with a spectral width of 20 MHz in the course of 2–3 d. The spectra, which at least partially exhibit features encoding structural information, are interpreted using a statistical model. This model finds a degree of MA–MA and FA–FA clustering ( 0.2≤S≤0.35 ). This proof-of-principle for both the wide-line NQR set-up and the statistical model widens the applicability of an underutilised avenue of non-invasive structural research.

## 1 Introduction

### 1.1 Wide-line nuclear quadrupole resonance (NQR)

The quadrupolar interaction occurs when a nucleus with spin quantum number I>1/2 is situated in a non-vanishing electric field gradient (EFG). In NMR, the resonance frequency of a quadrupolar nucleus depends on the orientation of this interaction with respect to the external magnetic field, resulting in specifically shaped powder patterns. The strength of the quadrupolar interaction determines the width of these patterns, and these can be tens or even hundreds of megahertz wide. Nuclear quadrupole resonance (NQR) [Bibr bib1.bibx10] probes the quadrupolar interaction directly in the absence of an external magnetic field. The stationary spin states are then the eigenstates of just the quadrupolar Hamiltonian. A detailed explanation of the associated energy level diagram can be found in publications such as [Bibr bib1.bibx31] and [Bibr bib1.bibx32]. Here, we just mention the case of spin I=5/2 and EFG asymmetry $\eta_{Q}\;=\;0$ , which is approximately true for all NQR studies reported in this work. This gives NQR transition frequencies

1 $$\nu_{n}\;=\;\frac{3n}{20}C_{Q},$$

where n=1,2 . In terms of the spin magnetic quantum number, these are the $\pm \frac{1}{2}↔\pm \frac{3}{2}$ and $\pm \frac{3}{2}↔\pm \frac{5}{2}$ transitions. Note that when $\eta_{Q}$ is not zero, these states mix, resulting in different frequencies and an additional allowed transition $\nu_{3}\;=\;\nu_{1}+\nu_{2}$ . In this example, as in all other NQR frequencies, there is no dependence on the orientation of the EFG and a linear dependence on $C_{Q}$ .

In well-ordered materials this results in sharp resonances. As the sensitivity improves with the strength of the interaction, NQR is particularly suitable where nuclear magnetic resonance (NMR) becomes impractical due to excessive spectral widths. When materials are disordered, however, the many unique local environments produce many different EFGs, each with their own resonance frequencies. Due to the sensitivity of NQR to structural variations, the spread of these signals can once again be several megahertz wide [Bibr bib1.bibx21]. While (1) this is still within reach of a proper wide-line set-up and (2) the lack of a powder pattern makes the spectrum significantly simpler than its NMR equivalent, the technical challenges and time investment of acquiring wide-line NQR spectra have so far limited its application.

### 1.2 Lead-halide perovskites

Over the last few decades, NQR has re-emerged from relative obscurity, in large part due to its usefulness in studying lead-halide perovskites (LHPs) [Bibr bib1.bibx37]. The exceptional properties of this class of materials have attracted attention for applications in liquid-crystal display technologies, light-emitting diodes, lasers, photodetectors and more [Bibr bib1.bibx29]. Perhaps the most intense interest comes from research into photovoltaics: over the last 15 years, perovskites in solar cells have gone from a sensitising dye yielding an efficiency of <3.81 % [Bibr bib1.bibx15] to inverted solar cells with an efficiency of >26.1 % [Bibr bib1.bibx5], establishing a role as an “emerging” photovoltaic material [Bibr bib1.bibx23].

Part of the appeal of LHPs lies in their capacity for being compositionally engineered. Perovskites are a family of crystals whose “ideal”, cubic structure is A ${}_{\left[m\bar{3}\;m\right]}^{\mathrm{XII}}$ B ${}_{\left[m\bar{3}m\right]}^{\mathrm{VI}}$ X ${}_{3[4/\mathrm{mm}]}^{\mathrm{II}+\mathrm{IV}}$ [Bibr bib1.bibx2], where the superscripts indicate the coordination number of each ion and the bracketed subscript the site symmetry. In lead-halide perovskites, B = Pb^2+^ and X = Cl^−^, Br^−^ or I^−^. Common occupants of the A site are Cs^+^ or small organic cations like formamidinium (FA^+^) or methylammonium (MA^+^). The latter are referred to as *hybrid* perovskites due to the mix of organic and inorganic components. All three sites can be made to contain a mix of ions to achieve certain benefits, such as a different bandgap [Bibr bib1.bibx22] or improved stability [Bibr bib1.bibx9]. Despite the promise of these mixed perovskites and a significant amount of previous research, there is still uncertainty regarding their structural and dynamic properties [Bibr bib1.bibx13], hindering the search for solutions to issues such as photoinduced halide segregation [Bibr bib1.bibx14] and instability of the perovskite structural phase [Bibr bib1.bibx6].

Due to the strong EFG between the lead ions and the large quadrupolar moments of the halides, their quadrupolar interactions are particularly strong, up to half a gigahertz for the iodides. This makes halide NQR particularly suitable for investigating the perovskite structure: it does not suffer from the excessive spectral broadening and convolution that hampers NMR, is not dependent on the presence of long-range order, and yet is very sensitive to local structure. However, its application has mostly been limited to LHPs with no or little mixing, such as FA_*x*_Cs_1−*x*_PbI_3_, where x≥0.9 [Bibr bib1.bibx1]. For more equal mixes, the width of the NQR spectrum is such that it once again poses a technical challenge to measure in its entirety.

### 1.3 Goal

Here, we seek to make wide-line NQR of disordered materials easily accessible. We demonstrate and validate a home-built, automated, variable-temperature wide-line NQR set-up. We employ an automated matching and tuning robot [Bibr bib1.bibx24] that, to the best of our knowledge, has only been used once before for NQR [Bibr bib1.bibx21], and we describe how we optimise the ease and quality of acquisition of automated measurements. We illustrate its usefulness with measurements of the second NQR resonance of ^127^I in mixed-cation LHP MA_*x*_FA_1−*x*_PbI_3_, where x=0.25,0.50 and 0.75 at a temperature range of 293–420 K . The second NQR resonance is chosen because it has a larger spin level population difference at equilibrium than the first resonance, yielding a better signal-to-noise (S / N) ratio. We show that it is possible to acquire variable-temperature, variable-offset cumulative spectra (VOCS) [Bibr bib1.bibx18] of hundreds of subspectra per day with an overall S / N of ≥100 , while requiring very little work from the operator.

The speed and ease of acquisition of wide-line NQR spectra that this set-up achieves enable a detailed investigation of the spectra of strongly mixed perovskites. We show a proof-of-concept version of such an analysis, to be refined and expanded upon in future work. On the phenomenological level, we construct a preliminary model that relates cation distributions to a spectral shape, to be fitted to experimental results. On a more fundamental level of theory, we perform density functional theory (DFT) calculations on structures provided by the molecular dynamics (MD) trajectory of MA_0.50_FA_0.50_PbI_3_ from [Bibr bib1.bibx13] and then compare observations with those of the phenomenological model.

Finally, we briefly showcase possible applications of the experimental set-up to related compounds, by measuring the mixed-anion and double-mixed perovskites MAPbI_2_Br and MA_0.15_FA_0.85_PbI_2.55_Br_0.45_. However, these are not subjected to modelling in this publication.

Taken together, we hope that these experimental and theoretical findings demonstrate the viability of this laboratory set-up as well as its potential to contribute to the structural investigation of disordered LHPs and similar materials.

## 2 Description of the models

The new laboratory set-up facilitates the acquisition of wide-line NQR spectra, but these are only useful if the structural information that they contain can be extracted. In order to demonstrate the potential of the set-up, we describe preliminary models for the interpretation of the spectra. Their purpose is to relate spectral features to nearest-neighbour ion substitutions and the degree of order in the overall ion distribution.

### 2.1 The phenomenological model

The phenomenological model is constructed as a function that can be fitted to the spectra. It directly simulates an NQR spectrum based on parameters that link particular configurations of the competing MA^+^ and FA^+^ cations in MA_*x*_FA_1−*x*_PbI_3_ to NQR resonance peaks. The model separates the compositional disorder following from the competing MA^+^ and FA^+^ cations in MA_*x*_FA_1−*x*_PbI_3_ into a short-range and a long-range component. The short-range component considers the NQR frequency and probability of a particular occupation of the lower cation coordination shells surrounding a halide. Each of these particular occupations, or “short-range coordinations”, correspond to a particular EFG. In the model, they are represented by a set of Lorentzians whose centre frequencies reflect their assumed EFGs and whose areas reflect their assumed probabilities.

For simplicity, coordinations with the same number of MA^+^ ions but different distributions are swept together. Following binomial statistics, the unbiased “fractional population” of a coordination in the n th shell containing $k_{n}$ MA^+^ cations and ( $N_{n}-k_{n}$ ) FA^+^ cations is then

2 pknn=Nnknxkn1-xNn-kn,

where $N_{n}$ is the total number of A-site cations in the n th coordination shell. Following [Bibr bib1.bibx35], a structural order parameter S [Bibr bib1.bibx7] is introduced that describes the tendency for coordination shells to be preferentially occupied by one type of cation. Equation ([Disp-formula Ch1.E2]) then becomes

3 pknn(S)=NnknxrMAkn1-rMANn-kn+1-xrFANn-kn1-rFAkn,

where

4rMA=x+S(1-x),5rFA=(1-x)+Sx.

S varies between 0, indicating a completely random distribution of the A-site cations, and 1, indicating complete phase segregation into MAPbI_3_ and FAPbI_3_. Intermediate values indicate partial clustering of cation species. The fractional population of a specific combination of coordination shells is just the product of the fractional populations per shell $\prod_{n}p_{k_{n}}^{n}(S)$ , where it is assumed that S is identical for all shells.

The NQR frequency of a particular short-range coordination is taken to be a “base” frequency plus offsets that scale linearly with the numbers of MA^+^ ions in the coordination shells. Mathematically, the frequencies are given by

6 $$\nu_{2}(\{k_{n}\})=\nu_{0}+\sum_{n}\Delta \nu_{\mathrm{MA}}[n]k_{n},$$

where $\Delta \nu_{\mathrm{MA}}[n]$ is the frequency shift per MA^+^ (instead of FA^+^) in the n th coordination shell of ^127^I and $k_{n}$ is the number of MA^+^ in this shell. Note again that these frequencies are independent of how the $k_{n}$ MA^+^ ions are distributed in their respective coordination shells.

The number of possible coordinations increases dramatically with the number of shells. At the same time, it is to be expected that shells at long range from the halide will have a small effect on the EFG. Therefore, anything beyond the first few shells is not considered explicitly; rather, it is accounted for as a separate long-range component of the model. A common and physically grounded approach to describe the influence of a large number of elements at long range on the EFG is the extended Czjzek distribution [Bibr bib1.bibx8], where the EFG of the short-range coordination serves as the local, fixed EFG in the extended Czjzek distribution. For a case in which the local EFG is sufficiently large and sufficiently symmetric, as is the case for the halides in perovskites, the NQR spectrum of an extended Czjzek distribution simplifies to a Gaussian distribution of the resonances associated with the local EFG. This reduces the simulation time of the model significantly.

To summarise, the phenomenological model consists of $4+n_{\max}$ parameters, where $n_{\max}$ is the number of shells considered in a short-range coordination, including

-
$\nu_{0}$ – the base frequency corresponding to zero MA^+^ ions in the short-range shells,
-
S – the order parameter,
-
$\Gamma_{L}$ – the full width at half maximum of the Lorentzian line broadening, and
-
$\Gamma_{G}$ – the full width at half maximum of the Gaussian expansion,

and $n_{\max}$ parameters $\Delta \nu_{\mathrm{MA}}[n]$ for the frequency shift per MA^+^ ion in n th shell of a short-range coordination.

Finally, experimental differences in intensities as a function of frequency and temperature must be taken into account. The intensity scales with the difference in Boltzmann equilibrium population between the energy levels involved in the resonance. The total simulated spectrum is scaled accordingly as a function of resonance frequency and temperature.

### 2.2 DFT-based models

The relation between short-range coordination and NQR frequency (Eq. [Disp-formula Ch1.E6]) is the cornerstone of the phenomenological model. It is an assumption whose validity cannot be confirmed by the phenomenological model itself; therefore, it should be verified using a higher level of theory. DFT calculations can be employed to study this relation independently. Molecular dynamics (MD) trajectories would provide a realistic model, but these are computationally expensive. The feasible lengths of such trajectories, and therefore their statistical accuracy, is poor. We present one such trajectory, but we add two simplified, although statistically stronger, models that focus on a specific interaction in the crystal lattice. All of these are based on the molecular dynamics (MD) trajectory described by [Bibr bib1.bibx13] of the simulation of a 4×4×4 supercell of MA_0.50_FA_0.50_PbI_3_ with random cation occupancy at 400 K . In each case, the EFG tensors of all 192 iodide ions in the supercells are calculated in the “lab” frame and then diagonalised to yield the EFG principal components and NQR frequency $\nu_{2}$ for each individual ^127^I ion.

The DFT-based models utilised are outlined in the following:

- *DFT-1*. In model DFT-1, we calculate the iodide EFG tensors on the MD trajectory for all iodide ions at regular intervals. For each I^−^, the temporal average of the EFG tensor on the MD trajectory is calculated (in the lab frame). This model accounts for all motion of all ions and is therefore, in principle, very realistic. As mentioned, however, the trajectories are rather short at 100 ps , and imperfect averaging of the tensors is expected.
- *DFT-2*. This simplified model probes the effect of the distortions in the inorganic backbone. To this end, Pb ions are placed at the time-averaged positions of the MD trajectory, with the iodide ions exactly halfway between nearest-neighbour Pb ions. The A-site cations are kept at their time-averaged positions as well, but they are replaced with Cs ions to minimise interactions based on cation species. This structure is not relaxed. The effect of the nature and shape of the cations on the iodide EFG tensors enters only indirectly via the positions of the Pb ions.
- *DFT-3*. Complementary to DFT-2, DFT-3 aims to assess only the effect of the presence of specific organic cations. The Pb ions are placed on an ideal cubic lattice whose lattice constant is determined by the average lattice parameters of the MD trajectory. The I^−^ ions are again fixed halfway in between. We model the cations as “effective” FA (MA) species occupying 12 (24) symmetry-equivalent positions in the Pb_8_I_12_ cubes: the effect of a single cation placement on the I^−^ tensors is determined by replacing all cations as present in the MD supercell by Cs ions, except for a single MA or FA cation. The 12 I^−^ of the cage around the organic cation are relaxed. The effect of one effective cation is obtained by averaging the EFG over the 12 (24) cation orientations with their corresponding distorted cages. In this way, we obtain the contribution to all iodide EFGs as a difference to a situation with only Cs cations. We loop over all cation sites and add the effect of each effective cation to all iodide tensors.

## 3 Methods

### 3.1 Density functional calculations

DFT calculations were carried out with the Vienna Ab initio Simulation Package (VASP; [Bibr bib1.bibx16]) using the projector augmented-wave (PAW) method [Bibr bib1.bibx3] and the Perdew–Burke–Ernzerhof (PBE) exchange-correlation potential [Bibr bib1.bibx25]. Electric field gradients (EFGs) were calculated using [Bibr bib1.bibx27]. The Brillouin zone was sampled with only the Γ point in a 4×4×4 supercell (or equivalent in smaller cells). The PAW data sets had frozen [Xe], [Kr] $4d^{10}$ , [Kr] $4d^{10}$ and $1s^{2}$ cores for Pb, I, Cs and C respectively. The ^127^I quadrupole moment was taken from [Bibr bib1.bibx30]. For the EFG calculations, the convergence threshold was $10^{-6}$ eV for the 4×4×4 supercell. Structural relaxations were carried out with a convergence threshold of $10^{-8}$ eV . The kinetic energy cutoff on the plane wave expansion was 500 eV .

### 3.2 Wide-line NQR set-up

NQR experiments were carried out using a home-built, single-channel probe based on a Chemagnetics probe housing with a horizontal seven-turn solenoid coil with a total length of 10 mm and an internal diameter of 5 mm made of 0.8 mm diameter (20-gauge) silver-plated copper wire. The temperature was regulated with a Chemagnetics temperature controller, calibrated with a thermocouple and confirmed by comparing the basal ^127^I $\nu_{2}$ NQR resonance frequency of MAPbI_3_ with [Bibr bib1.bibx41] up to 420 K . Tuning and matching was done by the H/F–X eATM robot from NMR Service GmbH [Bibr bib1.bibx24], adapted to the tuning and matching rods of the probe. Briefly, the ATM robot minimises the standing wave ratio (SWR) of a low-power continuous wave at the offset frequency of the subsequent acquisition. It does so by rotating the tuning and matching rods that would otherwise need to be adjusted by hand. A schematic representation of the complete variable-temperature, automatically tuned NQR set-up is shown in Fig. [Fig F1]. Using three easily replaceable capacitors, the set-up has a range of 140–182 MHz . Consistent power output across this range at high (150 W ) and low (10 mW ) power levels was calibrated using a Bird power meter and the eATM robot console respectively. An in-house Python script was employed to control frequency offsets, power levels, and the alternation of acquisition and matching and tuning during experiments and power calibration. Acquisition was done using a Varian VNMRS console and VnmrJ version 4.2 revision A. The external magnetic field strength at the probe location varied between 0.2 and 0.8 G depending on orientation. Both the location and orientation of the probe were kept constant throughout validation and experiments.

**Figure 1 F1:**
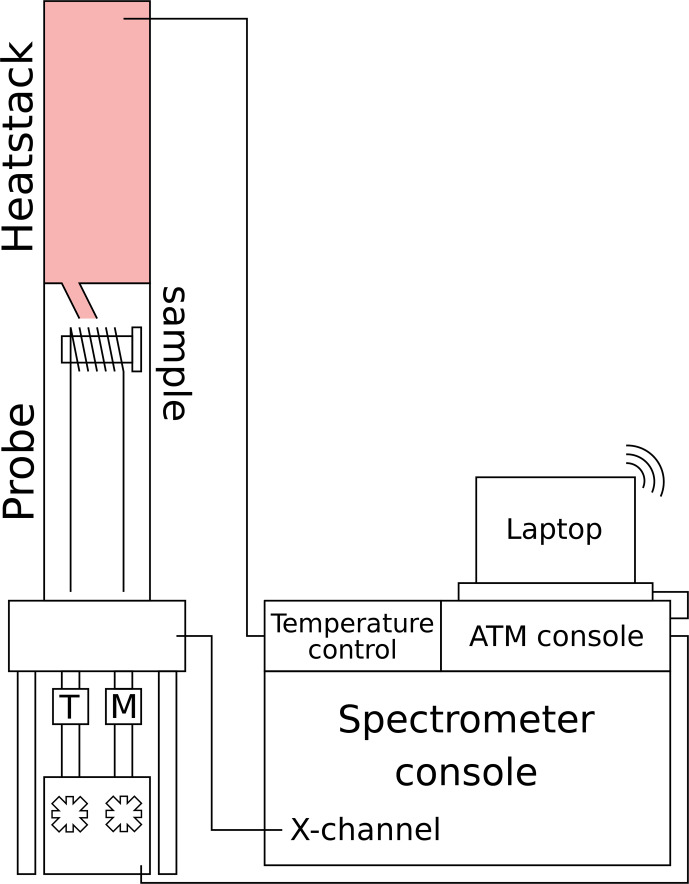
Schematic picture of the experimental set-up for NQR measurements of mixed-cation samples using the Varian VNMRS console, including temperature control and automatic matching and tuning. Control of the eATM robot is available through an internet connection to the eATM-dedicated laptop.

### 3.3 NMR and NQR measurements

Three samples were studied in detail: MA_0.75_FA_0.25_PbI_3_, MA_0.50_FA_0.50_PbI_3_ and MA_0.25_FA_0.75_PbI_3_. In addition, we acquired initial measurements of MAPbI_2_Br and MA_0.15_FA_0.85_PbI_2.55_Br_0.45_. All were synthesised through ball-milling of mixtures of methylammonium iodide (MAI), formamidinium iodide (FAI), methylammonium bromide (MABr), lead iodide (PbI_2_), lead bromide (PbBr_2_) or combinations of their perovskite products. These samples are the same as in [Bibr bib1.bibx13], who also describes the synthesis in more detail. These samples were stored in the dark under inert atmospheric conditions, while transfer to sample rotors was carried out under normal laboratory atmospheric and lighting conditions. For the MA_*x*_FA_1−*x*_PbI_3_ samples, the sample crystal structures were determined through X-ray diffraction to be the cubic I4/ mcm space group. After shipping, the ratios of the cations were confirmed by ^1^H MAS NMR to fall within 3 percentage points of the nominal ratios (see Table S2 in the Supplement). NMR experiments were performed using a MAGNEX 850 MHz magnet ( $B_{0}$ = 19.97 T ) equipped with a Bruker AVANCE NEO console and a Varian 3.2 mm HXY MAS probe in double-resonance mode. The ^1^H spectra were recorded using a one-pulse experiment in which the radio frequency (RF) field strength calibration and chemical shift referencing for all samples was done on powdered adamantane ( $\delta_{\mathrm{iso}}{(}^{1}H)\;$ = 1.756,1.873 ppm ). The spectra and acquisition parameters can be found in Sect. S1 in the Supplement. The samples were packed in Revolution NMR zirconia rotors.

**Table 1 T1:** Acquisition parameters for all ^127^I NQR spectra.

Parameter	Value
Temperature (K)	293, 300–420
Rotor diameter (mm)	5
Pulse sequence	Hahn echo VOCS
$\frac{\pi }{2}$ pulse length ( µ s)	1.8
π pulse length( µ s)	3.5
Echo delay ( µ s)	5
Recycle delay (ms)	6^a^
Spectral width (kHz)	2500
Excitation width (kHz)	225
VOCS step size (kHz)	50–200
Number of points	5000
Number of scans	$2^{14}$ – $2^{16^{b}}$

^a^ A value of 16 ms is used for MA_0.75_FA_0.25_PbI_3_ at 300 K . ^b^ A value of $2^{16}$ is used for room temperature measurements, whereas a value of $2^{14}$ is employed for 300–420 K measurements.

For the NQR experiments, the samples were packed in quartz tubes with an outer diameter of 5 mm . Teflon spacers were used to hold the sample in a volume of length 10 mm in the middle of the tube, to be placed in the middle of the RF coil for maximum RF homogeneity. Teflon caps were added to minimise the exposure of the sample to ambient atmosphere. All NQR spectra are recorded as VOCS [Bibr bib1.bibx18] consisting of Hahn-echo $\left(\frac{\pi }{2}\text{–}\tau -\pi \right)$ spectra for which both the $\frac{\pi }{2}$ - and π -pulse length were optimised for maximum signal strength on the 164.093 MHz ^127^I NQR resonance of MAPbI_3_. Using the same resonance, the full width at half maximum (FWHM) of the excitation profile of the Hahn echo was determined to be 225 kHz . More acquisition parameters can be found in Table [Table T1]. The range of detected frequencies was between 159 and 179 MHz for all MA_*x*_FA_1−*x*_PbI_3_ samples and between 140 and 175 MHz for MAPbI_2_Br and between 159 and 184 MHz for the doubly mixed MA_0.15_FA_0.85_PbI_2.55_Br_0.45_. In all cases, the entire range of frequencies of the second NQR resonance of ^127^I ( $\nu_{2}$ ) of the sample was covered. All processing was done in ssNake version 1.4 [Bibr bib1.bibx36]. Processing involved automated Lorentzian apodisation, zeroth-order phasing and conversion of data points to a common frequency axis.

**Figure 2 F2:**
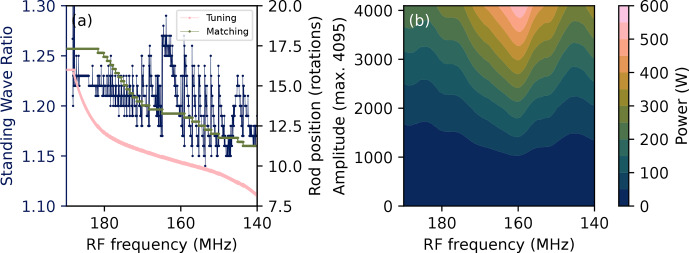
**(a)** Standing wave ratio and tuning and matching rod positions recorded during acquisition of the VOCS of an empty coil. **(b)** The power as a function of the RF frequency and the amplitude parameter in VnmrJ, recorded at 25 %, 50 %, 75 % and 100 % of the maximum amplitude. The underlying data are available from [Bibr bib1.bibx39].

## 4 Results and discussion

### 4.1 Wide-line NQR set-up optimisation

To show the validity of using the automated wide-line NQR set-up for quantitative measurements, the effective power as a function of offset frequency is characterised. Quantitative measurements require as little variation in the effective power as possible across the frequency range studied. The effective power can be affected by power reflected due to imperfect matching and tuning, the extent of which can differ as a function of offset frequency and depends on the probe. It is also not guaranteed that the power output of the spectrometer is constant over a large frequency range, but this can be compensated for by adjusting the power settings accordingly.

As part of every NQR VOCS acquisition, the reflected power is recorded in the form of the standing wave ratio by the eATM robot before the acquisition of each subspectrum. An example is shown in Fig. [Fig F2]a. The standing wave ratios of 1.15–1.30 correspond to a power reflection of 0.5 %–1.7 % across the frequency range. The upper frequency limit of the current configuration is determined by the edge of the matching capacitors at ∼17.5 rotations, and it was found to be ∼182.5 MHz . Below ∼140 MHz , the dip in the reflectance curves rapidly disappears entirely.

The power output of the spectrometer as a function of offset frequency is measured whenever any component of the set-up changes. Figure [Fig F2]b shows the relation between the VnmrJ amplitude parameter and the measured power output. Compensation for this inconsistency is integrated into the procedure for all measurements using the NQR set-up. It should be noted that the magnetic field amplitude of the pulse not only depends on the square root of the power but is also inversely proportional to the square root of the resonance frequency p. 247. However, the change is <1% in the region of 159–179 MHz that concerns the quantitative analysis of this work and was therefore not explicitly taken into account. However, this effect might be more substantial at lower frequencies and large bandwidth.

An important step in increasing the robustness of the VOCS acquisition involves the order in which the subspectra are recorded. For sufficiently small steps in the frequency offset $\Delta \nu_{\mathrm{RF}}\;<\;250$ kHz , the standing wave ratio is often below the threshold for automatic tuning to be executed, although nevertheless worse than before. The resultant periodic variation in reflection is visible as a shark's-tooth pattern in the skyline of the combined final spectrum. To counteract this, frequency-adjacent subspectra are decoupled by stepping back and forth between offsets that are more than 250 kHz apart. The list of n offsets $\nu_{\mathrm{RF},n}$ at which subsequent spectra are acquired are

7νRF,n=even=νRF,min+2n⋅ΔνRF,8νRF,n=odd=νRF,min+(1+m+2n)⋅ΔνRF,

where m is an even number such that $(1+m)\cdot \Delta \nu_{\mathrm{RF}}>250$ kHz .

Finally, two elements that further facilitate NQR experiments are worth mentioning. First, control of the tuning and matching robot, normally part of the same pulse sequence as the actual acquisition, is moved to a separate sequence, which is called by the external Python script between measurements. This removes the need to create a modified version of every pulse sequence one might want for acquisition. Second, the robot console is indirectly connected to the internet, allowing the operator to restart experiments, start new ones at different frequencies and even tune manually, all from a distance.

**Figure 3 F3:**
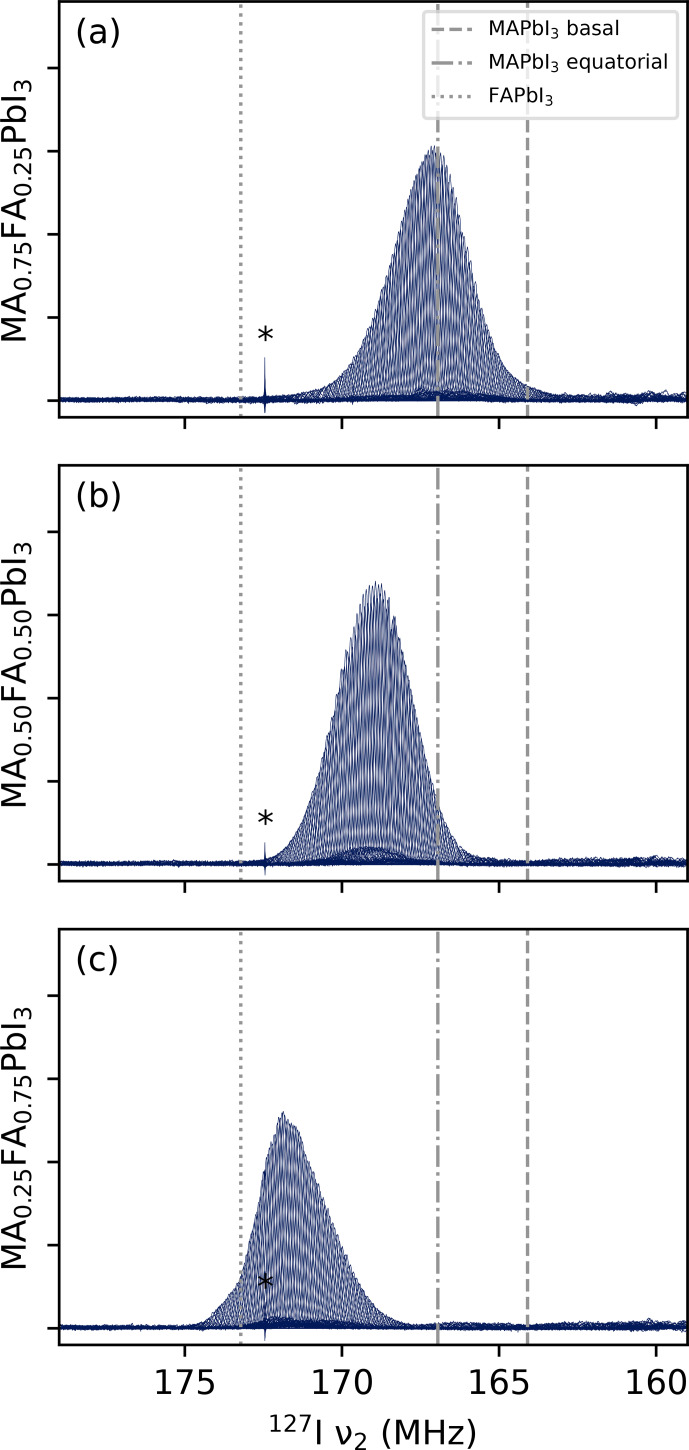
NQR VOCS at room temperature of **(a)** MA_0.75_FA_0.25_PbI_3_, **(b)** MA_0.50_FA_0.60_PbI_3_ and **(c)** MA_0.25_FA_0.75_PbI_3_. Dashed and dot-dash lines indicate the two resonances of the tetragonal MAPbI_3_. Dotted lines indicate the resonance of the cubic FAPbI_3_ as taken from [Bibr bib1.bibx41]. All spectra show a sharp feature around 172.5 MHz , indicated by an asterisk, presumed to be some sort of external radio signal. Intensities should not be compared between spectra due to small differences in experimental conditions. The roughness of the skyline in panel **(b)** is the shark's-tooth pattern described in Sect. [Sec Ch1.S4.SS1]; this pattern is not observable in panels **(a)** and **(c)** and easily compensated for during processing. The underlying data are available from [Bibr bib1.bibx39].

### 4.2 ^127^I NQR at room temperature

The complete set of VOCS at room temperature for the three LHP samples are shown in Fig. [Fig F3]. A total of 400 subspectra could be recorded over the course of a weekend with, for MA_0.50_FA_0.50_Pb_3_, an overall S / N ratio of ∼400 . Attention required from the operator was limited to a little surveillance. By comparison, earlier attempts to record these spectra manually and with less optimisation required around 30 min per subspectrum and involvement from the operator at every frequency change.

As can be expected based on the resonance of the unmixed compounds (indicated in the figure), the resonances shift to higher frequencies with increasing formamidinium content. Note that this is accompanied by an increase in the lattice constant [Bibr bib1.bibx38]. At fixed composition, such a lattice expansion is expected to decrease the resonance frequency, as the slope of the electric field decreases with distance between the charges. Here, however, some property of the formamidinium evidently counteracts and outweighs this effect. In addition, the spectra appear “skewed” towards the pure resonance of its majority cation, with MA_0.50_FA_0.50_PbI_3_ being completely symmetrical. This a common phenomenon in binomial distributions and, therefore, not surprising.

Less expected, and therefore more interesting, is the difference in shape between the peaks of MA_0.75_FA_0.25_PbI_3_ and MA_0.25_FA_0.75_PbI_3_. While the former is fairly smooth, the latter exhibits (reproducible) features, particularly at the top of the peak. These features, as well as the fact that they are not mirrored in a compound with opposite cation ratios, show that even extremely broad NQR spectra are a source of information concerning the disorder in the lattice, if properly interpreted.

### 4.3 ^127^I NQR at increased temperatures

Figure [Fig F4] shows the spectra of the three perovskite samples at different temperatures. In these and following figures, only the point of highest intensity per VOCS subspectrum is shown. For all spectra, an increase in temperature corresponds to a shift to lower frequency and a decrease in intensity. The former is consistent with thermal expansion increasing the distance between ions involved in the EFG. The decrease in intensity is mostly caused by the smaller spin state population differences at thermal equilibrium at higher temperatures and lower frequencies. To compensate for this effect, the intensity of each data point can be divided by the population difference at thermal equilibrium of two energy levels whose energy separation corresponds to the frequency of the data point. The integrated intensity after this operation is shown in Fig. [Fig F5]a. There remains no clear relation between temperature and signal strength or peak FWHM (Fig. [Fig F5]b) for MA_0.25_FA_0.75_PbI_3_ or MA_0.50_FA_0.50_PbI_3_. For MA_0.75_FA_0.25_PbI_3_, the intensity decreases and the FWHM increases, pointing towards increased relaxation.

**Figure 4 F4:**
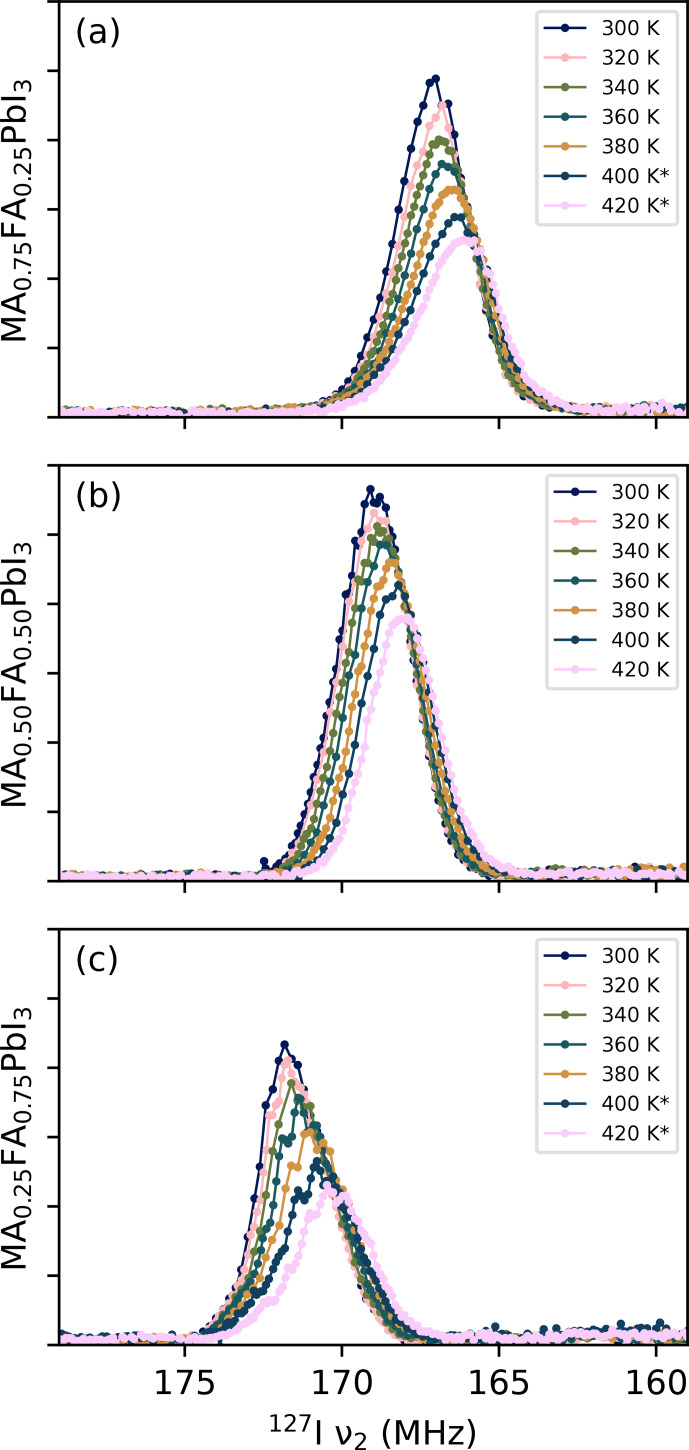
Variable-temperature NQR VOCS for **(a)** MA_0.75_FA_0.25_PbI_3_, **(b)** MA_0.50_FA_0.50_PbI_3_ and **(c)** MA_0.25_FA_0.75_PbI_3_. Starred spectra are more detailed repeat measurements. Their intensities have been scaled to match the original measurements at that temperature. The underlying data are available from [Bibr bib1.bibx39].

**Figure 5 F5:**
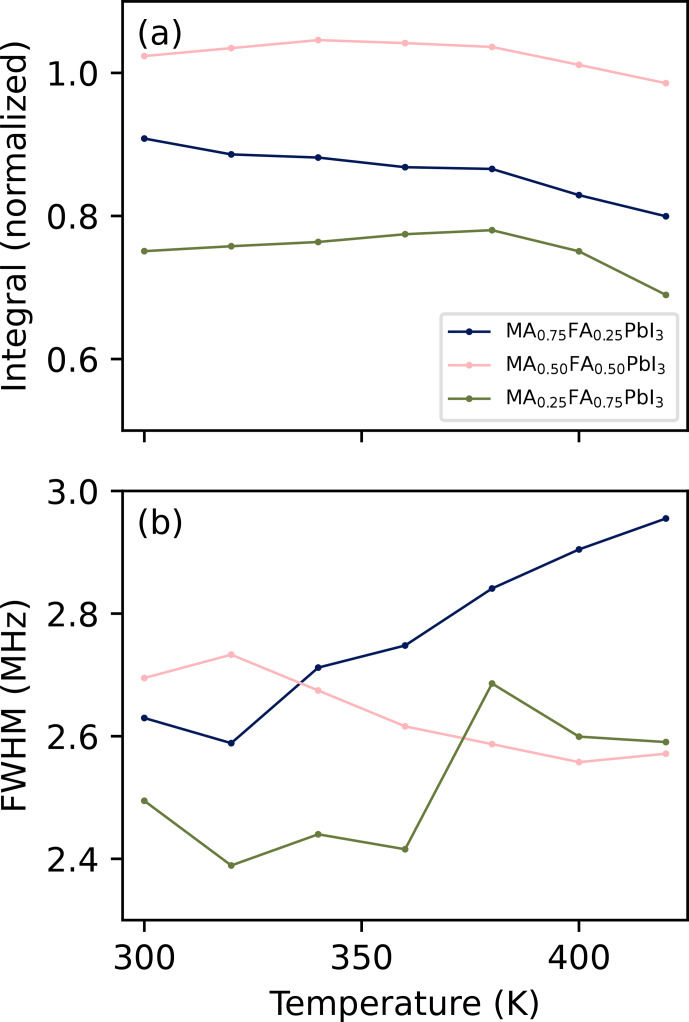
Properties of the spectra in Fig. [Fig F4] after scaling intensities to compensate for temperature and frequency. **(a)** Integrals over the scaled intensities, normalised to the total integral of an identically acquired VOCS of MAPbI_3_ at 320 K (see Fig. S2 of the Supplement). **(b)** Full width at half maximum of the scaled spectra. The underlying data are available from [Bibr bib1.bibx39].

It is interesting to see how the shapes of the peaks in Fig. [Fig F4] change with temperature. The spectrum of MA_0.75_FA_0.25_PbI_3_ becomes less symmetric (it increasingly skews to low frequencies), whereas that of MA_0.25_FA_0.75_PbI_3_ becomes more symmetric and that of MA_0.50_FA_0.50_PbI_3_ stays symmetric. These shape changes indicate a temperature-dependent property in the material that itself depends on the cation composition, which could, for example, be a temperature-dependent degree of local order. It is also noteworthy that the spectral features of MA_0.25_FA_0.75_PbI_3_ become more pronounced at higher temperatures. While this probably plays a role in the irregular trend in Fig. [Fig F5]b, it also points to some temperature-dependent property. These observations, along with those in Sect. [Sec Ch1.S4.SS2], illustrate the information present in NQR spectra of these kind of samples. The extraction of information, however, requires novel models.

### 4.4 Wide-line NQR modelling

#### 4.4.1 The phenomenological model

The great compositional disorder of these cation mixes drives the spectral shape towards featureless humps. This particularly holds for MA_0.50_FA_0.50_PbI_3_ which, combined with the symmetry of its cation mix, looks very much like a single Gaussian. There is no point in applying the model proposed in Sect. [Sec Ch1.S2.SS1] here, as there will be many solutions of similar quality but wildly different parameter values. Fortunately, fits of the spectral shape of the other two samples, especially MA_0.25_FA_0.75_PbI_3_, are particular enough to the model parameters that some observations can be made.

**Figure 6 F6:**
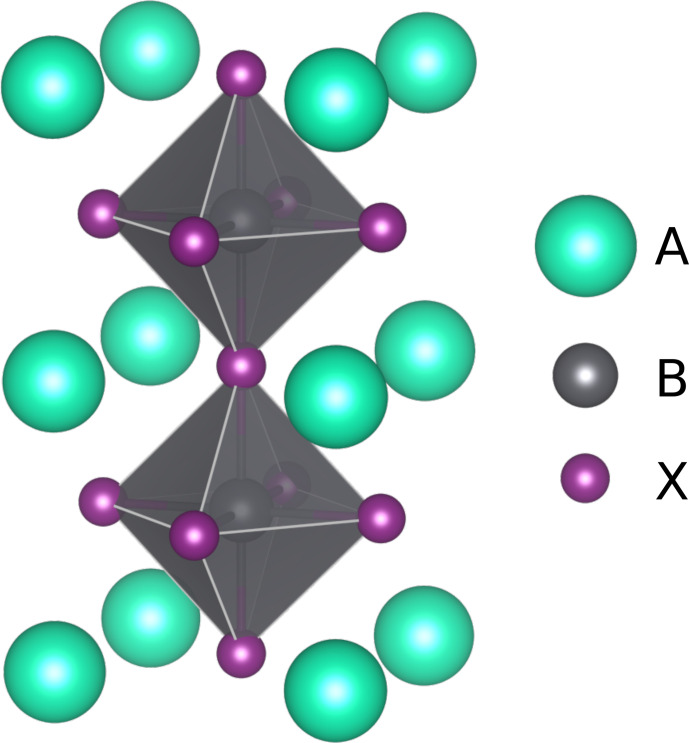
The cubic perovskite crystal structure including the first and second shells of A-site cations with respect to the X -site anion. Image made with VESTA [Bibr bib1.bibx20].

The first and strongest observation concerns the relative influence of short-range coordination shells of A-site cations. At 420 K , the spectrum of MA_0.25_FA_0.75_PbI_3_ shows more than five peaks. As the first shell around an iodide consists of four A-site cations (see Fig. [Fig F6]), this is more than the five possible MA : FA ratios of this shell. Clearly, at least one more coordination shell has to have a distinctive influence on the resonance frequencies. The phenomenological model (Sect. [Sec Ch1.S2.SS1]) therefore needs to include two shells in its short-range coordinations. With the addition of the eight A-site cations of the second shell, the model consists of 45 peaks.

**Figure 7 F7:**
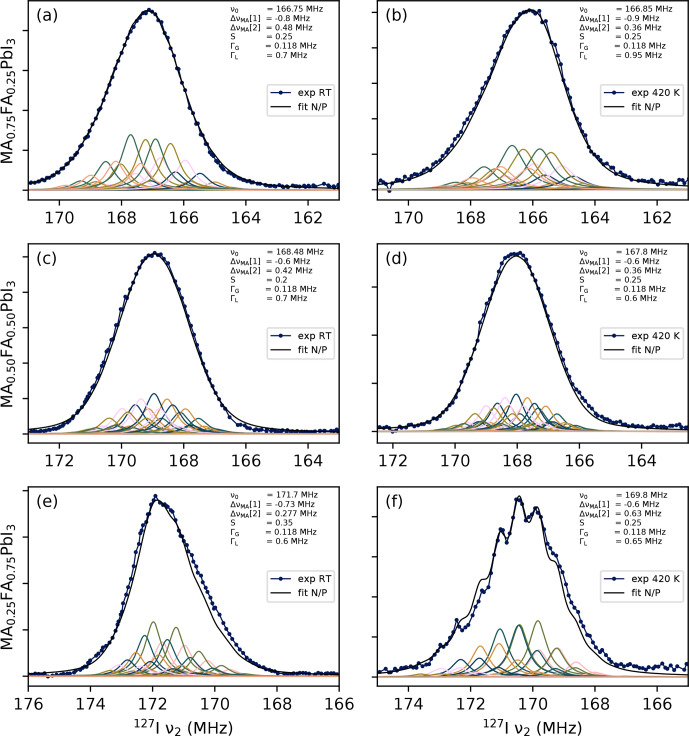
Manual fits of all mixed-cation samples at room temperature **(a, c, e)** and at 420 K **(b, d, f)**. The shifts per first- or second-shell MA, indicated as $\Delta \nu_{\mathrm{MA}}[1]$ and $\Delta \nu_{\mathrm{MA}}[2]$ respectively, are constrained to being negative or positive (N/P).

**Table 2 T2:** Manually determined optimal fitting parameters of mixed-cation perovskite ^127^I NQR spectra at low and high temperature under the constraint that the shift per first-shell MA $\Delta \nu_{\mathrm{MA}}[1]$ is negative and the shift per second-shell MA $\Delta \nu_{\mathrm{MA}}[2]$ is positive. The underlying data are available from [Bibr bib1.bibx39].

	MA75		MA50		MA25	
Temperature (K)	293	420	293	420	293	420
$\nu_{0}$ (MHz)	166.75	166.85	168.48	168.40	171.70	169.80
$\Delta \nu_{\mathrm{MA}}[1]$ (MHz)	- 0.80	- 0.90	- 0.60	- 0.60	- 0.73	- 0.60
$\Delta \nu_{\mathrm{MA}}[2]$ (MHz)	0.480	0.360	0.420	0.360	0.277	0.630
S	0.25	0.25	0.20	0.25	0.35	0.25
Gauss (MHz)	0.1	0.1	0.1	0.1	0.1	0.1
Lorentz (MHz)	0.70	0.95	0.70	0.60	0.60	0.65

The second observation concerns the sign of the frequency shift per MA in the first ( $\Delta \nu_{\mathrm{MA}}[1]$ ) and second shells ( $\Delta \nu_{\mathrm{MA}}[2]$ ). It is assumed that these signs are consistent between samples. The fits are denoted by their combination of signs, with P for positive and N for negative; for example, a fit where $\Delta \nu_{\mathrm{MA}}[1]>0$ and $\Delta \nu_{\mathrm{MA}}[2]<0$ is labelled P/N and is part of the P/N “submodel”. Manual fits of all four submodels have been made for all three samples, at room temperature and at 420 K . The parameters of these fits are tabulated in Sect. S3. The N/P submodel is the only one with which qualitatively satisfactory fits could be realised for all of these spectra, in the sense that the relative intensities of the subpeaks and the overall shape are qualitatively reproduced. These fits are shown in Fig. [Fig F7] (some examples of unsatisfactory fits can be found in Figs. S3 and S4). In other words, it appears that replacing an FA^+^ cation with an MA^+^ ion will decrease the EFG when done in the first coordination shell, whereas it will increase the EFG when done in the second shell. The exact fitting parameters are also tabulated in Table [Table T2].

Finally, the N/P fits include values for the order parameter S . As seen in Fig. [Fig F7], 0.2<S<0.35 . This is consistent with results from [Bibr bib1.bibx13], who measured dipolar couplings between protons of neighbouring MA and FA ions and concluded that 0.2<S<0.4 for MA_0.25_FA_0.75_PbI_3_ and MA_0.50_FA_0.50_PbI_3_, whereas 0.0<S<0.4 for MA_0.75_FA_0.25_PbI_3_. Note that this describes the same samples. Therefore, the aforementioned work and this study both suggest that there is a modest tendency for cation species to cluster together.

It should be noted that any quantitative information, including the order parameter, should be interpreted with care. These fits were done manually, and the relation between parameters and spectral shape is fairly complex. Although an effort was made to explore different sections of the parameter space, there is no real guarantee that there are no equivalent fits with significantly different values. The observations regarding the number of relevant shells and the signs of $\Delta \nu_{\mathrm{MA}}[1]$ and $\Delta \nu_{\mathrm{MA}}[2]$ are more reliable, however, as they depend on qualitative features of the spectra that other versions of the model inherently fail to reproduce. The exact values of these parameters (see Table [Table T2]) should again be interpreted cautiously, although the fit for MA_0.25_FA_0.75_PbI_3_ at 420 K demonstrates how the presence of highly visible subpeaks can indicate a case in which $\Delta \nu_{\mathrm{MA}}[1]\approx -\Delta \nu_{\mathrm{MA}}[2]$ .

### 4.5 DFT-based models

The first-principles models described in Sect. [Sec Ch1.S2.SS2] provide an independent perspective of the nature of $\Delta \nu_{\mathrm{MA}}[1]$ and $\Delta \nu_{\mathrm{MA}}[2]$ . They each produce a distribution of 192 ^127^I frequencies that can be subdivided according to the number of first-shell ( $k_{1}$ ) or second-shell ( $k_{2}$ ) MA^+^ ions. An overview is presented in Fig. [Fig F8], and the full result can be found in Sect. S4. It should be noted that the statistical accuracy is limited, particularly for $k_{2}=0$ , 1, 7 and 8.

**Figure 8 F8:**
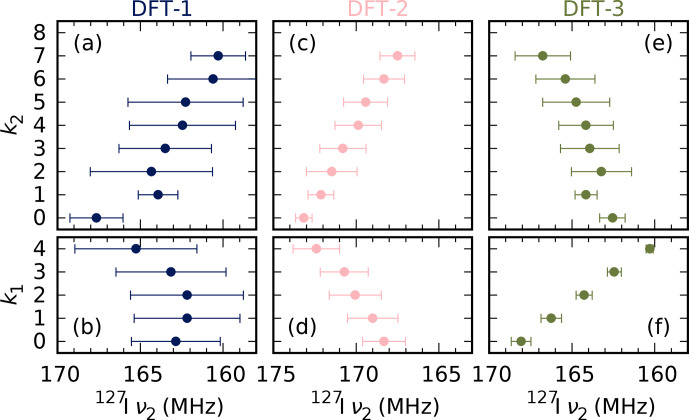
Average and root-mean-square values of the simulated ^127^I NQR $\nu_{2}$ resonances in the models described in Sect. [Sec Ch1.S2.SS2], as a function of the number of MA^+^ ions in the first ( $k_{1}$ ) and second coordination sphere ( $k_{2}$ ). Note that the number of data points n is quite limited: n= 12, 48, 66, 60 and 12 for $k_{1}\;=$ 0–4 and n= 2, 2, 24, 38, 62, 32, 28, 4 and 0 for $k_{2}\;=$ 0–8.

The model that is, in principle, most realistic, DFT-1 (Figs. [Fig F8]a–b, S5), fails to show a connection between cation coordination and frequency. The frequencies are not converged, however, as not all of the relevant combinations of orientations of the 12 surrounding cations of each ^127^I have been sampled. Longer simulation would likely yield narrower spectra and, we hope, better-resolved temporal averages and clear trends, but these are computationally very expensive.

The simpler models DFT-2 and DFT-3 provide some insight at a fraction of the cost. They still produce quite broad frequency distributions, but some trends can be identified. In model DFT-2 (Figs. [Fig F8]c–d, S7), more MA^+^ in the first (second) shell increases (decreases) the resonance frequency. In the terminology of the previous section, it has a clear P/N trend. This is correlated with the Pb–Pb distance, which is inversely related to the frequency (see also Fig. S6). This is consistent with the experimentally confirmed decrease in NQR frequencies with thermal expansion [Bibr bib1.bibx41]. The complementary model DFT-3 (Figs. [Fig F8]e–f, S8) has the opposite trend (N/P). Evidently, the local effect of the cations on the anions (model DFT-3) counteracts the indirect effect from distorting of the inorganic backbone (model DFT-2). The effects are roughly comparable in magnitude, so it is not clear what the overall effect would be in more realistic models.

In summary, the simplified DFT models identify a phenomenon consistent with the findings of the phenomenological model but fail to confirm or reject them. To do so requires longer, expensive MD trajectories after all. It is our hope that work in this field makes these trajectories considerably more feasible. A sufficiently long MD trajectory would refine and constrain the phenomenological model, improving both its realism and ease of use.

### 4.6 ^127^I NQR of other ion mixes

Finally, Fig. [Fig F9] gives a preview of the type of spectra to be expected from different types of mixed perovskites. Below the bandwidth of 159–179 MHz tried and tested so far, spectra exhibit spectral distortions that are attributed to the transgression of frequency-dependent limits in the transmitter/receiver electronics. It will be necessary to adjust or further increase the frequency range of the probe to study these kinds of compositions. However, it is already apparent that a mix of halides gives rise to a significantly broader spectrum than that of mixed A-site cations. After scaling intensities to compensate for the temperature, frequency and iodine content, the total integral of MAPbI_2_Br and MA_0.15_FA_0.85_PbI_2.55_Br_0.45_ is 0.8 and 0.9 times that of MA_0.25_FA_75_PbI_3_ respectively or 0.6 and 0.65 that of MAPbI_3_ at 320 K respectively. Given the roughness of the spectra, these numbers should be taken as overestimates. They confirm the trend from Fig. [Fig F5] that mixing ions can lead to significant signal loss. In addition, the full width at half maximum is more than 7 MHz in both spectra, almost 3 times that of the mixed-cation samples. Still, Fig. [Fig F9] establishes that NQR studies of these perovskites variants are possible, pending the elimination of low-frequency distortions).

**Figure 9 F9:**
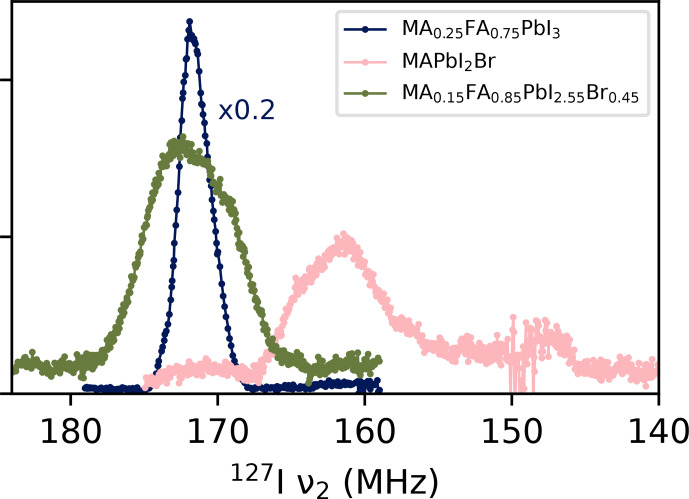
Room temperature NQR VOCS for various kinds of mixed-ion perovskites. All spectra were acquired with $2^{16}$ scans per subspectrum, and the samples were of comparable weight ( ±10% ). Below ∼153 MHz , spectra exhibit distortions tentatively attributed to the transgressions of limits of the probe circuitry. The underlying data are available from [Bibr bib1.bibx39].

## 5 Conclusions

We have realised and demonstrated a laboratory set-up making wide-line NQR faster and easier, and we have applied this to obtain spectra of ^127^I in mixed-ion lead-halide perovskites. We acquired spectra with great signal-to-noise ratios at various temperatures, enabling detailed studies of these compositionally disordered materials. We show that the spectra of methylammonium–formamidinium mixed LHPs hold a great potential for elucidating local structure and dynamics. Preliminary modelling suggests that cation substitution in the first shell around the halide has an effect on the electric field gradient on the halide opposite to cation substitution in the second shell. Simple DFT models point to two competing mechanisms, currently preventing confirmation of the overall trend. We also identify a degree of local order consistent with previous research ( 0.2<S<0.35 ). Proving these hypotheses requires additional research. More extensive MD calculations will be necessary to provide clearer support for the phenomenological model. In addition, the new set-up allows for easy acquisition of additional compositions that will permit the identification of clear trends.

Quickly acquired and interpretable NQR spectra should open the way towards new experiments, including (but not limited to) in situ measurements that are complicated to achieve in normal NMR. The combination of broadband NQR probes and automated matching and tuning can also be very useful for other materials, not just for very broad spectra but also in cases where the NQR resonance is not yet known. Furthermore, it can be useful in the acquisition of NQR spectra broadened by Zeeman perturbation.

## Supplement

10.5194/mr-6-143-2025-supplement The supplement related to this article is available online at https://doi.org/10.5194/mr-6-143-2025-supplement.

## Supplement

10.5194/mr-6-143-2025-supplement
10.5194/mr-6-143-2025-supplement The supplement related to this article is available online at https://doi.org/10.5194/mr-6-143-2025-supplement.

## Data Availability

The raw data and processing steps of the NQR and NMR spectra described in this publication, as well as the NQR calibration data, are stored on a repository maintained by Radboud Universiteit (10.34973/cwk8-we61, [Bibr bib1.bibx39]) and are publicly available.

## Appendix

The supplement related to this article is available online at https://doi.org/10.5194/mr-6-143-2025-supplement.
