# Supplementary material for: Automated wide-line nuclear quadrupole resonance of mixed-cation lead-halide perovskites
Source: Magn Reson (Gott). 2025 Jul 16;6(2):143–55. doi: 10.5194/mr-6-143-2025 (PMC12285786; doi:10.5194/mr-6-143-2025)
Supplement: The supplement related to this article is available online at https://doi.org/10.5194/mr-6-143-2025-supplement. [file mr-6-143-2025-supplement.pdf]

Supplement of Magn. Reson., 6, 143–155, 2025
https://doi.org/10.5194/mr-6-143-2025-supplement
© Author(s) 2025. CC BY 4.0 License.

Supplement of

Automated wide-line nuclear quadrupole resonance of mixed-cation lead-halide perovskites

Jop W. Wolffs et al.

Correspondence to: Arno P. M. Kentgens (a.kentgens@nmr.ru.nl)

The copyright of individual parts of the supplement might differ from the article licence.

S1. ^{1}H and ^{1}H→^{13}C MAS NMR of $MA_xFA_{1-x}Pb_3$

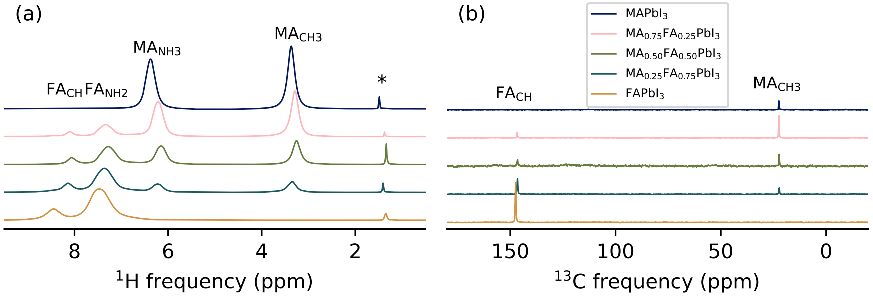

Figure S1: (a) ^{1}H MAS and (b) ^{1}H→^{13}C CPMAS spectra of $MA_xFA_{1-x}Pb_3$ ($x = 1.00, 0.75, 0.50, 0.25, 0.00$ from top to bottom). The asterisk in the ^{1}H MAS spectrum indicates a cyclohexane impurity resulting from wet-milling synthesis. Number of scans and intensity scaling varies, especially for the ^{1}H→^{13}C CPMAS spectra. Acquisition parameters are listed in Table S1. The underlying data are available at Wolffs et al. (2025).

Table S1: Acquisition parameters the ^{1}H and ^{1}H→^{13}C CPMAS NMR spectra of Fig. S1

Parameter	^{1}H	^{1}H→^{13}C
Magnetic field (T)	20.0	20.0
Temperature	RT	RT
rotor diameter (mm)	3.2	3.2
Pulse sequence	one pulse	CPMAS
^{1}H rf field strength 90° pulse (kHz)	99	99
^{1}H rf field strength during contact (kHz)	-	74
^{13}C rf field strength during contact (kHz)	-	60
Contact time (ms)	-	10
Contact ramp shape (%)	-	90-100
^{1}H rf field strength during decoupling (kHz)	-	75
Decoupling scheme	-	SPINAL
Recycle delay	$(5\times T_1)^{a}$	$(5\times T_{1,^1\mathrm{H}})^{a}$
Spectral width (kHz)	200	100
Spinning frequency (kHz)	15	15
Number of points	40000	6000–8000
Number of scans	16–32	256–512

[a] 50, 85, 80, 75 and 160 seconds for $x = 1.00, 0.75, 0.50, 0.25, 0.00$ respectively.

Table S2: Stoichiometries of the mixed samples based on ^{1}H and ^{13}C NMR, both from this work and the previous owner of the samples, Grüninger et al. (2021). The underlying data are available at Wolffs et al. (2025).

	^{1}H MAS		$^{1}H \rightarrow {}^{13}C$ CPMAS	
Sample	This work	Grüninger et al.	This work	Grüninger et al.
$MA_{0.75}FA_{0.25}Pb_3$	0.75:0.25	0.75:0.25	0.78:0.22	0.75:0.24
$MA_{0.50}FA_{0.50}Pb_3$	0.51:0.49	0.49:0.51	0.50:0.50	0.48:0.52
$MA_{0.25}FA_{0.75}Pb_3$	0.25:0.75	0.25:0.75	0.25:0.75	0.75:0.74[a]

[a] Taken literally from Grüninger et al. but presumably this is supposed to be 0.25:0.74.

S2. $MAPbI_3$ VOCS

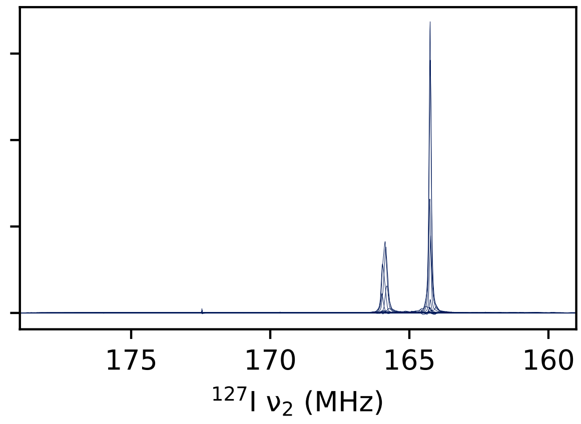

Figure S2: NQR VOCS at 320 K of $MAPbI_3$. Fitting the summed spectrum with three peaks (two at ~166 MHz) yields Gaussian broadening of < 0.08 MHz and Lorentzian broadening of < 0.12 MHz.

S3. ^{127}I NQR fitting parameters

Table S3: Manually determined optimal fitting parameters of mixed cation perovskite ^{127}I NQR spectra at low and high temperature under the constraint that both the shift per first shell MA $\Delta\nu_{\mathrm{MA}}[1]$ and the shift per second shell MA $\Delta\nu_{\mathrm{MA}}[2]$ are negative. The underlying data are available at Wolffs et al. (2025).

	MA75		MA50		MA25	
Temperature (K)	293	420	293	420	293	420[a]
ν_0 (MHz)	172.35	171.45	171.7	170.55	172.90	171.60
$\Delta\nu_{\mathrm{MA}}[1]$ (MHz)	-0.500	-0.650	-0.550	-0.500	-0.550	-0.600
$\Delta\nu_{\mathrm{MA}}[2]$ (MHz)	-0.600	-0.520	-0.412	-0.375	-0.440	-0.540
S	0.100	0.300	0.250	0.300	0.325	0.325
Gauss (MHz)	0.1	0.1	0.1	0.1	0.1	0.1
Lorentz (MHz)	0.95	0.80	0.70	0.60	0.60	0.60

[a] Fits at these temperatures are very poor.

Table S4: Manually determined optimal fitting parameters of mixed cation perovskite ^{127}I NQR spectra at low and high temperature under the constraint that the shift per first shell MA $\Delta\nu_{\mathrm{MA}}[1]$ is positive and the shift per second shell MA $\Delta\nu_{\mathrm{MA}}[2]$ is negative. The underlying data are available at Wolffs et al. (2025).

	MA75		MA50		MA25	
Temperature (K)	293	420	293	420	293[a]	420[a]
ν_0 (MHz)	167.85	168.30	169.48	168.40	171.90	171.00
$\Delta\nu_{\mathrm{MA}}[1]$ (MHz)	0.75	0.55	0.60	0.60	0.50	0.65
$\Delta\nu_{\mathrm{MA}}[2]$ (MHz)	-0.488	-0.605	-0.420	-0.390	-0.450	-0.585
S	0.30	0.25	0.20	0.20	0.35	0.00
Gauss (MHz)	0.1	0.1	0.1	0.1	0.1	0.1
Lorentz (MHz)	0.70	0.90	0.70	0.60	0.60	0.65

[a] Very poor fits.

Table S5: Manually determined optimal fitting parameters of mixed cation perovskite ^{127}I NQR spectra at low and high temperature under the constraint that both the shift per first shell MA $\Delta\nu_{\mathrm{MA}}[1]$ and the shift per second shell MA $\Delta\nu_{\mathrm{MA}}[2]$ are positive. The underlying data are available at Wolffs et al. (2025).

	MA75		MA50		MA25	
Temperature (K)	293[a]	420[a]	293	420	293[a]	420
ν_0 (MHz)	162.10	161.00	166.10	165.25	170.35	168.55
$\Delta\nu_{\mathrm{MA}}[1]$ (MHz)	0.65	0.60	0.60	0.60	0.60	0.60
$\Delta\nu_{\mathrm{MA}}[2]$ (MHz)	0.455	0.540	0.420	0.390	0.450	0.660
S	0.3	0.000	0.200	0.175	0.000	0.150
Gauss (MHz)	0.1	0.1	0.1	0.1	0.1	0.1
Lorentz (MHz)	0.70	0.95	0.70	0.70	0.60	0.70

[a] Very poor fits.

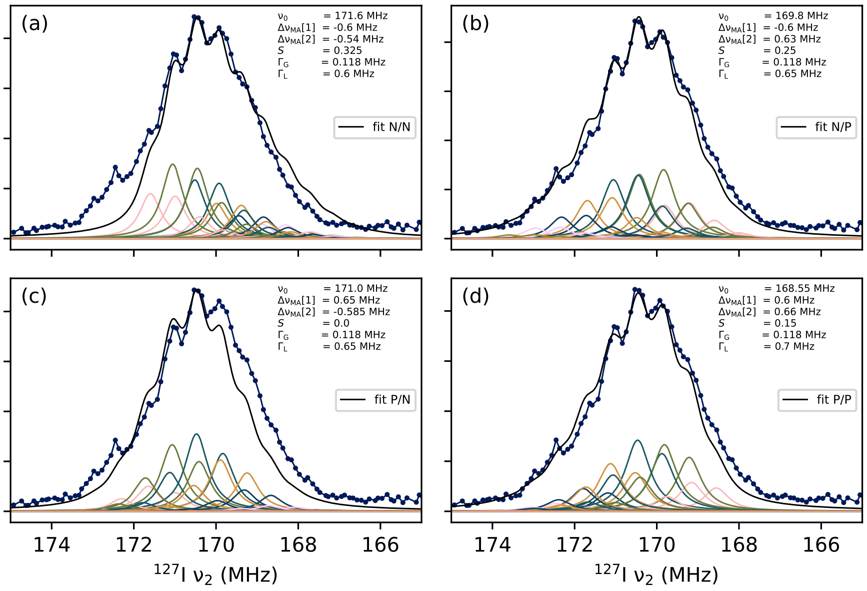

Figure S3: Manual fits of $MA_{0.25}FA_{0.75}PbI_3$ at 420 K. The shifts per first/second shell methylammonium $\Delta\nu_{\mathrm{MA}}$ [1]/[2] are constrained to being positive (P) or negative (N): (a): N/N, (b): N/P, (c): P/N, (d): P/P.

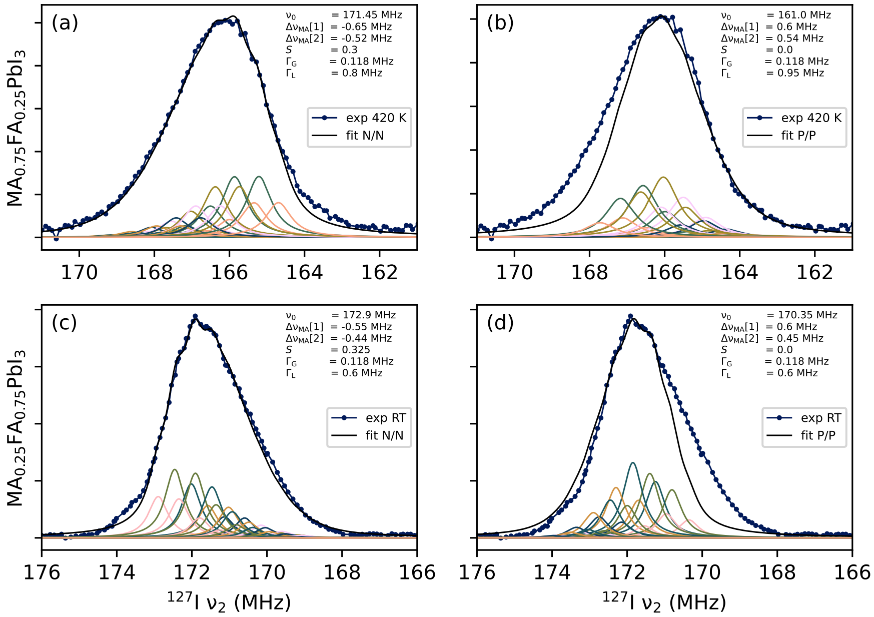

Figure S4: Manual fits of high temperature $MA_{0.75}FA_{0.25}PbI_3$ (a,b) and room temperature $MA_{0.25}FA_{0.75}PbI_3$ (c,d). The shifts per first/second shell MA $\Delta\nu_{\mathrm{MA}}$ [1]/[2] are constrained to being positive (P) or negative (N): (a,c): N/N, (b,d): P/P.

S4. Quantum chemical calculations

Detailed results of the ^{127}I NQR ν_2 resonance simulations using the four models from Sec. 2.2. The spectra were modelled as a convolution with a Gaussian with standard deviation $\sigma = 0.1$ MHz.

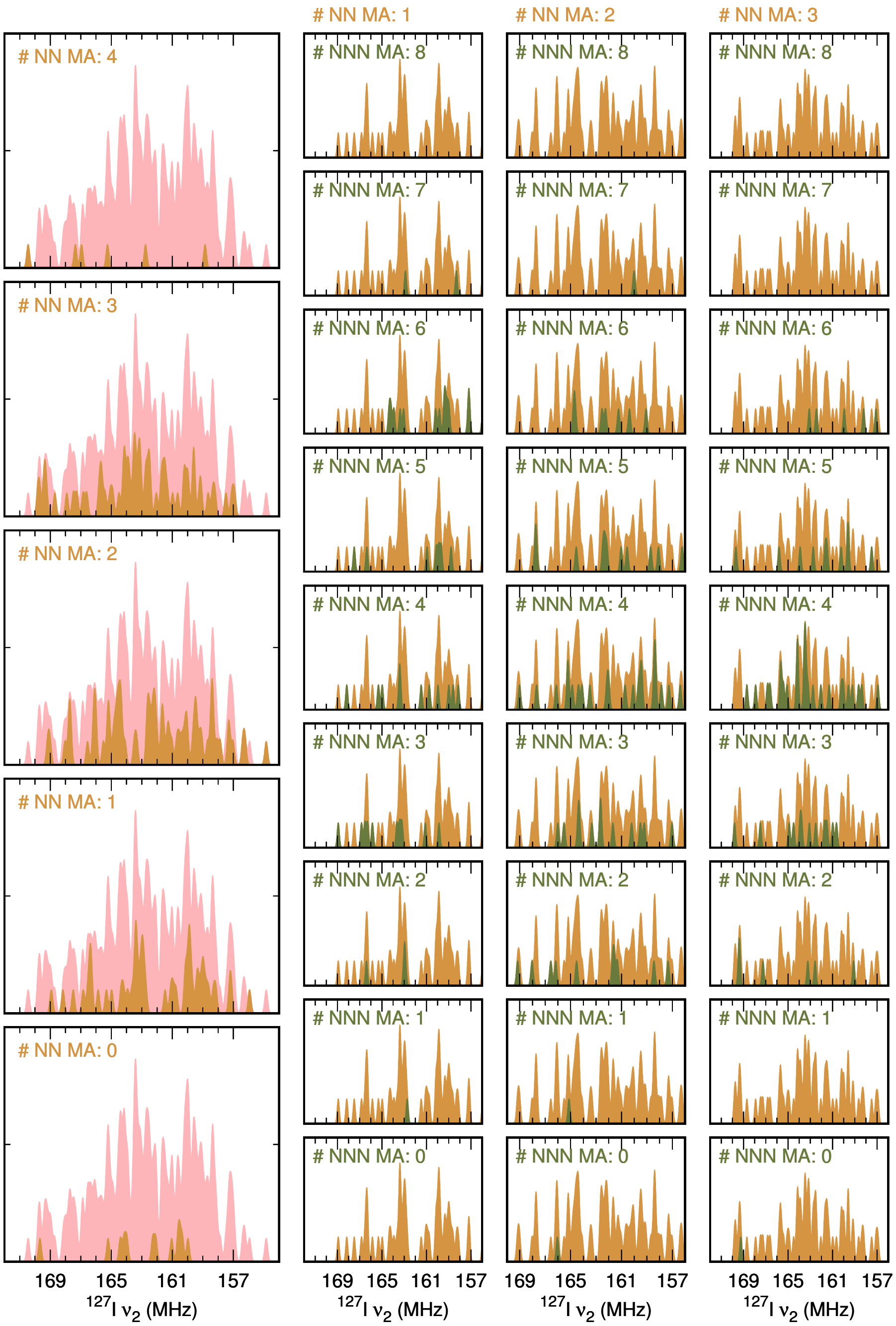

Figure S5: MD-predicted ^{127}I v_2 distributions of $MA_{0.50}FA_{0.50}PbI_3$ at 400 K, using a $4 \times 4 \times 4$ unit cell, i.e., model **DFT-1**. The distribution for all 192 ^{127}I is pink, the distributions with fixed number of nearest neighbour (NN) MA are in yellow. Column 2 (3) [4] shows the distribution for varying number of next nearest neighbour (NNN) MA (in green) for a single (two) [three] MA NN.

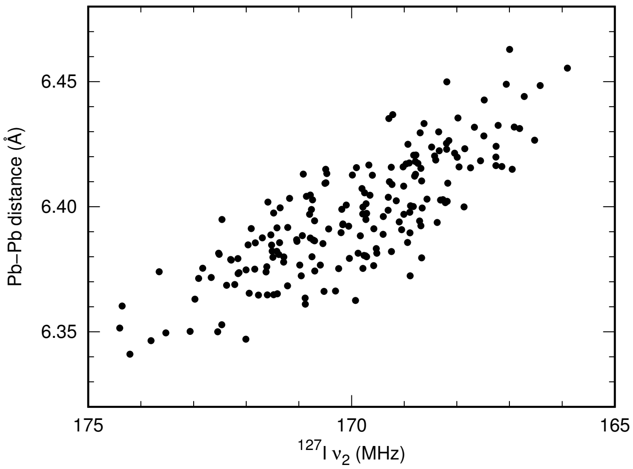

Figure S6: Scatter plot of Pb-Pb distances and coorsponding ^{127}I v_2 of the iodide bridging the two Pb ions using the time-averaged model **DFT-2**.

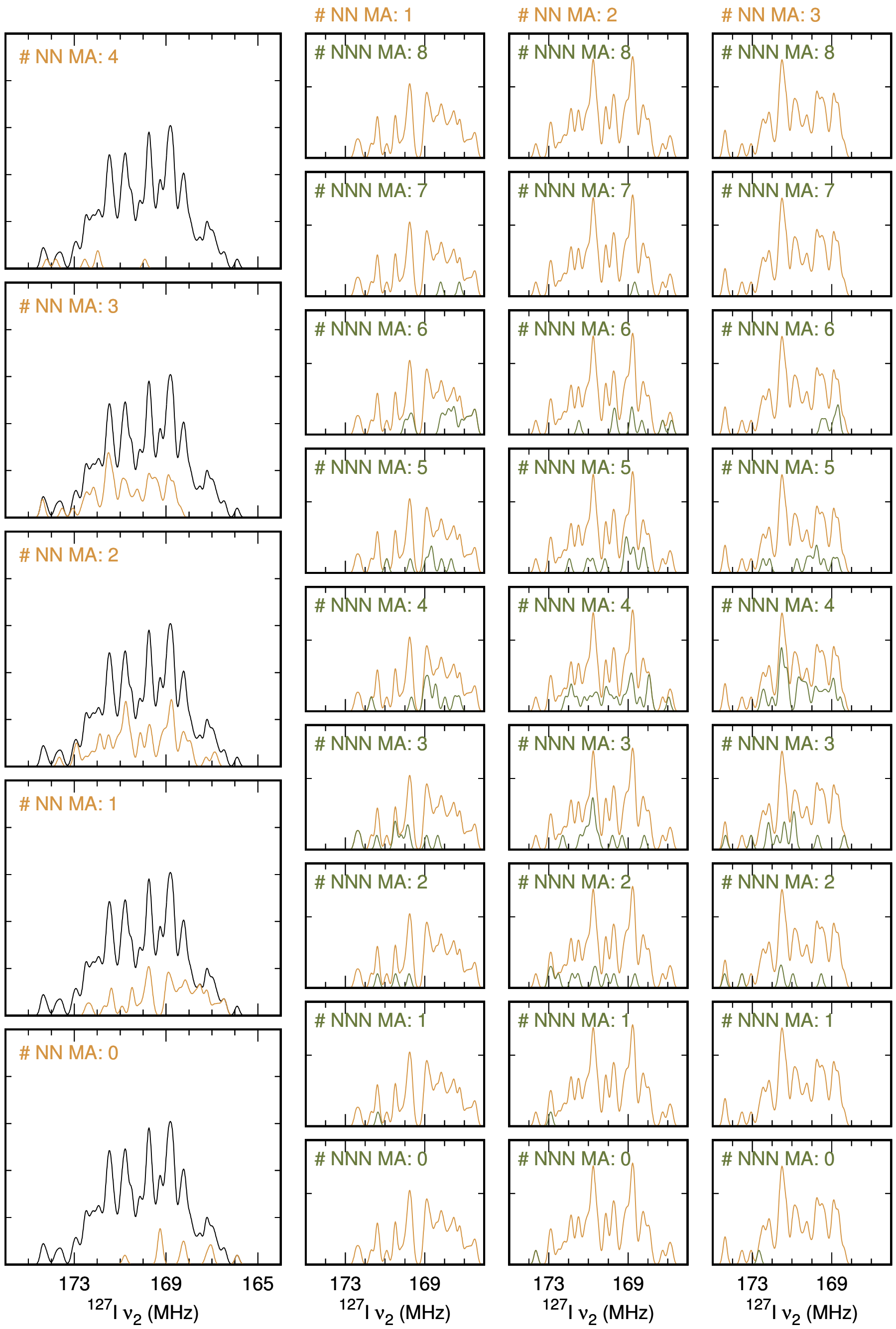

Figure S7: Distributions of ^{127}I v_2 according to **DFT-2** (cf. Fig. S5)

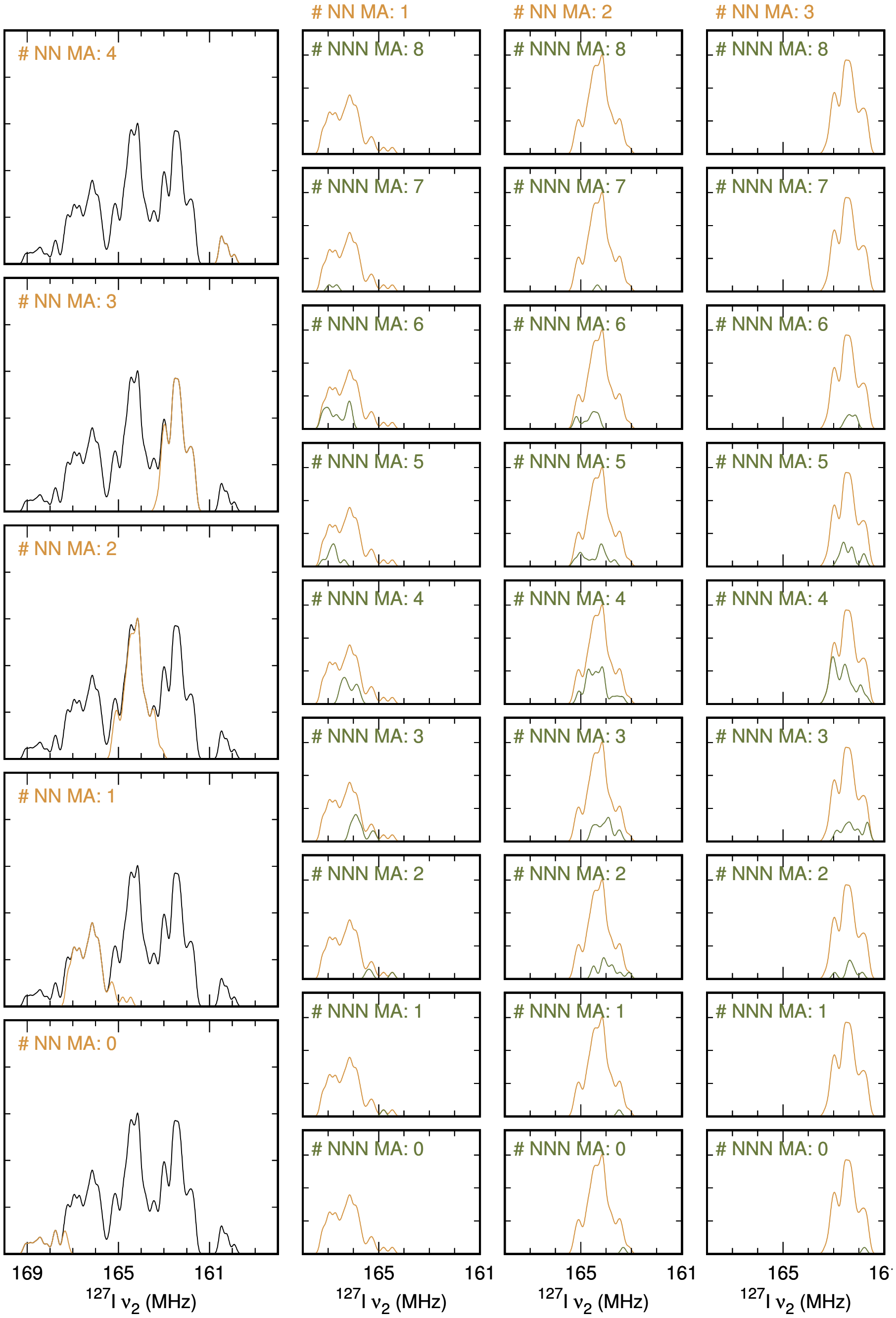

Figure S8: Distributions of ^{127}I v_2 according to **DFT-3** (cf. Fig. S5)

References

Grüninger, H., Bokdam, M., Leupold, N., Tinnemans, P., Moos, R., De Wijs, G. A., Panzer, F., and Kentgens, A. P. M.: Microscopic (Dis)order and Dynamics of Cations in Mixed FA/MA Lead Halide Perovskites, The Journal of Physical Chemistry C, 125, 1742–1753, https://doi.org/10.1021/acs.jpcc.0c10042, 2021.

Wolffs, J. W., Gómez Badillo, J. S., Janssen, G. E., De Wijs, G. A., and Kentgens, A. P.: Automated wideline nuclear quadrupole resonance of mixed-cation lead halide perovskites, https://doi.org/https://doi.org/10.34973/cwk8-we61, 2025.
